# AndroMalPack: enhancing the ML-based malware classification by detection and removal of repacked apps for Android systems

**DOI:** 10.1038/s41598-022-23766-w

**Published:** 2022-11-14

**Authors:** Husnain Rafiq, Nauman Aslam, Muhammad Aleem, Biju Issac, Rizwan Hamid Randhawa

**Affiliations:** 1 Department of Computer and Information Sciences, Northumbria University, Newcastle upon Tyne, UK; 2 Department of Computer Sciences, National University of Computer and Emerging Sciences, Islamabad, Pakistan

**Keywords:** Computer science, Information technology, Software

## Abstract

Due to the widespread usage of Android smartphones in the present era, Android malware has become a grave security concern. The research community relies on publicly available datasets to keep pace with evolving malware. However, a plethora of apps in those datasets are mere clones of previously identified malware. The reason is that instead of creating novel versions, malware authors generally repack existing malicious applications to create malware clones with minimal effort and expense. This paper investigates three benchmark Android malware datasets to quantify repacked malware using package names-based similarity. We consider 5560 apps from the Drebin dataset, 24,533 apps from the AMD and 695,470 apps from the AndroZoo dataset for analysis. Our analysis reveals that 52.3% apps in Drebin, 29.8% apps in the AMD and 42.3% apps in the AndroZoo dataset are repacked malware. Furthermore, we present AndroMalPack, an Android malware detector trained on clones-free datasets and optimized using Nature-inspired algorithms. Although trained on a reduced version of datasets, AndroMalPack classifies novel and repacked malware with a remarkable detection accuracy of up to 98.2% and meagre false-positive rates. Finally, we publish a dataset of cloned apps in Drebin, AMD, and AndrooZoo to foster research in the repacked malware analysis domain.

## Introduction

Android operating system (OS) dominates the smartphone industry with more than 85% global market share^1^ becoming the prime target for malware developers. Industry and researchers are paying significant attention to securing smartphone devices. The research community has proposed various solutions to analyze and avoid the hazards caused by malware^2,3^. However, a torrent of Android malware attacks (over 12 million) has emerged in the recent past^4^. Most of the time, attackers produce clones by repacking existing legitimate or malicious apps to achieve the desired malevolent objectives^5^. According to some previous studies^6^, 80% of mobile malware is repackaged. Since the Android apps are available to download from public app stores, an attacker can easily retrieve the legitimate app, reverse engineer and inject malicious code into it. The attacker can later publish the modified version of the original app on a public app store^7^. This kind of attack refers to a repackaging attack. The motivation behind the app repackaging is not always malicious. It has been observed that some developers get access to the source code of premium apps, repack and distribute the cloned versions for free, which refers to application plagiarism. The plagiarized version of premium apps is further used as a source of income by incorporating paid advertisements and in-app purchases.

Numerous techniques have been proposed to detect Android repackaged malware^8^. Machine learning (ML) being the core element of Android malware detection, most of the techniques discussed in^8^ focus on detecting the clones. However, to our knowledge, no previous study has investigated the effects of removing repackaged apps from training datasets. The classification results of ML algorithms highly depend on the quality of the data used for the training process. However, pre-processing the training data can be a time-consuming task. In the case of Android, the apps need to be reverse-engineered to extract the features. Various tools are used to reverse engineer the Android apps^9^, whereas the time required for the reverse engineering process depends on the app’s size. Since 2015, Google has increased the size limits on Android apps from 50 to 100 MB^10^, and with the growth of the apps, the cost of reverse engineering could increase even further. Moreover, the training and optimization time required for the ML algorithms also depends on the size of the training dataset. Consequently, the repackaged apps in the training sets of ML algorithms result in increased costs.

This paper first highlights the problem of repackaged malware by finding the potential clones of existing malware in three benchmark Android malware datasets. In order to quantify the occurrence of repacked malware in the datasets, we match the package names of samples under observation with those of known malicious packages. Then, we investigate the impact of cloned apps based on the same package names on multiple machine learning models. We name our proposed technique as AndroMalPack, which extracts permissions, APIs and Intent-based features from the apps dataset to train the machine learning models. AndroMalPack removes all the repacked malware samples (based on package name reusing) from the training set. However, it retains the repacked malware in the test sets to measure the effectiveness of ML models. AndroMalPack employs seven different machine learning models (support vector machine (SVM), linear regression (LR), decision trees(DT), random forests (RF), xgboost (XGB), AdaBoost (AB) and k-nearest neighbours (KNN) ) with default hyper-parameters trained on the clones free train-sets. Moreover, AndroMalPack selects the best performing ML model on reduced datasets and tunes the hyper-parameters by employing nature-inspired algorithms (NIAs) to achieve even better results. Three nature-inspired algorithms (bat, firefly and grey wolf optimizer) are used to optimize the hyper-parameters of the best performing classifier. Finally, we publish a comprehensive dataset of cloned apps based on the same package names in Drebin, AMD and AndoZoo datasets to support further research in repacked malware analysis.

The key contributions of this work can be summarized as follows:

1. We quantify the potential clones of known malware in 3 benchmark Android malware datasets (Drebin, AMD and Androzoo) by employing a lightweight and novel strategy based on package names reusing.
2. We propose AndroMalPack, a novel approach for Android malware classification. AndroMalPack is trained on clones free data and optimized using nature inspired algorithms. Contrary to traditional 80/20 train and test splits, AndroMalPack filters outs the repacked malware (based on package name reusing) from training sets, whereas test sets contain all repacked malware in addition to non-repacked and benign samples. Consequently, AndroMalPack significantly reduces the training set size yet retains high classification accuracy. Although trained on reduced train sets, AndroMalPack outperforms multiple state of the art techniques in terms of classification results.
3. We publish a hash dataset of 389,995 repackaged apps based on package names reusing in Drebin, AMD and Androzoo repositories to foster future research in repacked Android malware analysis domain.

The rest of the paper is organized as follows. Section “Background” covers the background concepts, and Section “Motivation” presents the motivation for this work and research questions. Section “Datasets” presents the details of datasets used and the statistics about potential repacked malware based on package names reusing. Section “AndroMalPack” presents AndroMalPack, an Android malware classifier trained on clones free train sets and optimized using nature-inspired algorithms. We then present the experimental results in Section “Experimental results”. We discuss the related work and comparison with state of the art in Section “Related work”. Section “AndroMalPack dataset” presents details about the published dataset, and we conclude our work in Section “Conclusion”.

## Background

This section discusses some background about Android application (APK) structure, APK reverse engineering, APK repackaging, and the motivation behind this study.

### Android application structure

Android applications are usually developed using Java programming language and are deployed in a compressed form called the Android application package (APK). APKs can be downloaded and installed directly on Android devices from the official app store called Google play or from third-party app stores like the Amazon app store, GetJar and Opera app store. A typical Android APK consists of the following components:

1. *Dalvik byte code* Android applications are written in Java and are further compiled in the form of *.class* files (Dalvik byte code). The *.class* files are then compressed in the form of a single Dalvik executable file called *classes.dex* which is finally executed on the Dalvik virtual machine (DVM).
2. *Manifest file* Every Android application has an *AndroidManifest.xml* file which contains essential information about the components and structure of the app. The *AndroidManifest.xml* file includes the information about the main package name, permissions that the app requires, hardware components which the app accesses, activities, broadcast receivers, services, and intent filers and the software features required by the app.
3. *Resources* it includes all the essential resources required by the app like images, animations, layouts and user interface strings. All the required resources are compiled into the app at the built time.
4. *Libraries* contains all compiled external libraries which are used in the app.
5. *Signatures* The author must digitally sign all the Android applications before deployment on app stores. The digital signature is a unique cryptographic hash which represents the author.

### Reverse engineering

**Figure 1:**
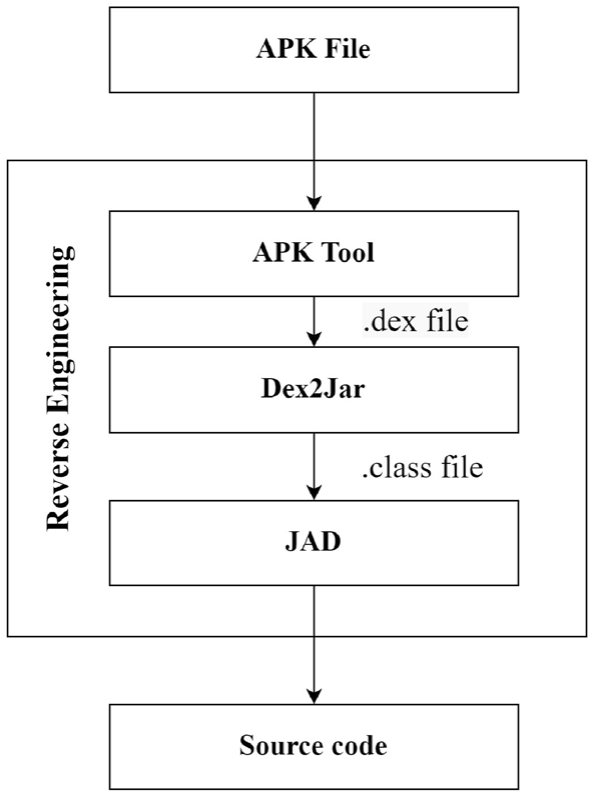
Android application package (APK) reverse engineering.

Android application package (APK) is a zipped archive that contains *classes.dex* file, *AndroidManifest.xml* file, resources and libraries in compressed form. The contents of the zip archive are not human-readable as the java classes are compressed in the form of a single Dalvik executable (*.dex*) file. However, it is possible to reverse engineer an APK file to extract java source code and corresponding files using several tools. Figure 1 explains the steps required to reverse engineer an APK. APK tool is used to unzip the APK file to extract *classes.dex* file. The *classes.dex* file is then decompiled in form of java-archive *.jar* file by using *dex2jar* tool. The *.jar* produced by *dex2jar* tool contain java byte code in form of *.class* files which are still not human readable. Finally, a java decompiler tool such as JAD is used to decompile the *.class* files in the form of java source code.

### Android application repackaging

Application repackaging refers to reverse engineering an app, injecting custom functionality, and re-assembling the app into deployable form. Malware developers commonly use application repacking to inject malicious payloads into cloned versions of popular apps on the Android platform. Malware developers often repack existing malware to evade antivirus systems. Most antivirus systems depend on the signatures of known malware for malware detection^11^. The malicious signatures databases of the antivirus systems are updated regularly. In the case of Android, a simple unpack and recompilation of the application without any modifications results in a change of the entire signature^12^. Re-assembling the app changes the organization of contents like classes, methods and variables in the *classes.dex* file, which eventually affects the signature of the app. Consequently, attackers regularly use the practice of simple recompilation to create exact clones of known malware to deceive antivirus systems. Apart from malicious code injection and simple repackaging, attackers also repack premium apps with custom advertisements and distribute them for free to generate revenue.

### Application naming conventions

Every Android app available on the app store must have an app name and a unique package name. App name refers to the app’s title that appears on the app store. It is not requisite for an app to have a unique name as multiple apps on the Android official app store can be found sharing the same name. On the other hand, the Android package name is the unique identity of an app which is defined in *AndroidManifest.xml* file. Usually, the package name is the name of the base package, which is created when the app is developed. The base package can have further sub-packages containing java classes and activities. No two apps installed on the same device can have identical package name^13^. If two apps with identical package names are installed on the same device, the latter will override the previous one as an updated version. Malware authors frequently upload cloned apps with the same package names and slight modifications to trick antivirus systems which rely on hash-based detection.

## Motivation

Our preliminary study on repacked malware started with an investigation of malware samples from the Drebin dataset^14^. Drebin contains 5560 malware samples belonging to 117 different malware families. To detect repacked malware, we selected 1793 malware samples from the top 5 families based on the number of samples in each family (Table 1). Furthermore, we reverse-engineered the selected apps to extract multiple features like permissions, intents, hardware components, the network address and package names. Interestingly, we found a massive redundancy amongst the apps’ package names under analysis. Our findings reveal that 48.68% of the apps in the selected dataset share some frequently used package names. Consequently, we churned out the apps which share the same package names for further analysis. As discussed earlier, simple re-compilation of Android apps (re-construction of *classes.dex* file) results in a significant change in the app’s signature. Therefore, all the apps that share the same package names still have different hash values, and as a result, a more robust signature generation technique is needed. Our target at this stage was to develop a novel signature generation technique such that all the samples that have the same package names should have identical signatures. Subsequently, instead of relying on calculating the hash value of *classes.dex* file, we considered the hash generation for all the extracted source code of the apps.

**Table 1:** Malware samples in Drebin from Top 5 families.

Malware family	Samples
FakeInstaller	821
OpFake	363
BaseBridge	330
Kmin	147
FakeDoc	132

Further analysis of apps sharing the same package names revealed that most share the same source code with minor changes. Traditional hash generation algorithms like SHA1^15^ and MD5^16^ take input from a file of arbitrary size and produce a fixed-length cryptographic hash as an output. Calculating the SHA1 or MD5 hash of two identical files will always produce the same output. Most antivirus systems maintain contemporary databases of MD5 and SHA1 hashes of know malware. However, a minor change in the original malware results in a significant SHA1 or MD5 hash change. Therefore, instead of calculating SHA1 or MD5 hashes of the source codes of the apps sharing the same package names, we considered using a more robust hashing technique called SSDeep^17^. SSDeep is based on a context-triggered piece-wise hashing (CTPH) technique known as fuzzy hashing. CTPH is a powerful new technique that can detect homologous files, i.e., almost identical files. Given the fuzzy hashes of two almost identical files, i.e. the original file and a file with some minor changes, the ssdeep algorithm can provide the similarity score between two hashes. Conversely, SHA1 and MD5 hashes do not have the capability of comparing the similarity between two hashes. Therefore, we considered using fuzzy hashes. If there are any minor changes in the cloned malware, we still can get a similarity score by comparing it with know malware hashes.
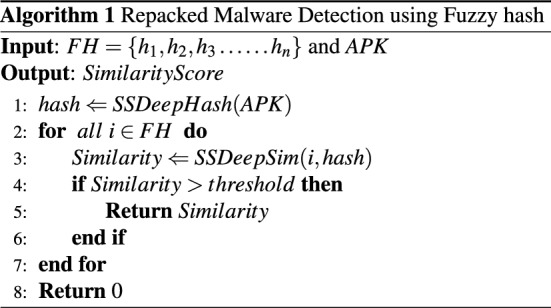


Algorithm 1 presents our fuzzy hash-based methodology to detect repacked malware. Let *D* be the dataset of the top 5 families from the Drebin dataset. We reverse engineer all the apps in *D* to extract a set of distinct package names as $DPN=\{Pn_{1}, Pn_{2}$, $Pn_{3}, . . . Pn_{n}\}$. Furthermore, we randomly select one app form *D* for each package name in *DPN*, calculate its fuzzy hash using SSDeep algorithm and place it in a set *FH*. The set of fuzzy hashes *FH* and an APK from *D* are provided as input to the Algorithm 1, whereas a similarity score is produced as an output. We calculate the fuzzy hash of the source code of the given APK as the first step *(Algorithm 1, line 1)*. The hash of the APK is then compared with all the hashes in *FH* by using the hash comparison utility of SSDeep algorithm *(Algorithm 1, line 3)*. If the similarity score at any point is greater than the threshold value, the APK is declared as repacked malware, and the similarity score is returned *(Algorithm 1, lines 4–6)*. The algorithm returns 0 if none of the hashes in *FH* has a similarity score above the threshold. The threshold value for experiments was set at 70% similarity score.

Table 2 summarizes the results of repacked malware detection by using fuzzy hashes. We used 873 malware samples from 5 families for experiments and found six frequently reused packages. Furthermore, we randomly selected 1 sample from each set of apps sharing the same package name and calculated its fuzzy hash. The fuzzy hash is then compared with hashes of all the remaining samples, which share the same package names. The app is declared repacked malware if its fuzzy hash has a 70% similarity score with any of the hashes in *FH*. As reported in Table 2, the average detection rate based on fuzzy hashes of malware samples sharing the same package name is 58.81%. Although the results from fuzzy hash-based detection are not promising, however, provided us with solid motivation for further analysis of repacked malware based on package name reusing. Further in this work, instead of focusing on signature-based detection, we employ machine learning-based algorithms to detect repacked malware. We further extend the scope of our work by employing another two Android malware datasets to investigate malicious apps sharing identical package names. Based on the results of the motivating study, we focus on addressing the following research questions further in this study:

- RQ1: How often do malware samples in benchmark Android malware repositories re-use package names?
- RQ2: Can we consider the malware samples sharing same package names as clones/repacked versions of known malware?
- RQ3: What is the impact of repacked malware on ML-based Android malware classifiers?

**Table 2:** Fuzzy Hash-based similarity results.

Package name	Family	Samples	Similarity
com.software.application	FakeInstaller	234	10.6%
com.software.appinstaller	FakeInstaller	193	66.8%
com.keji.danti	BaseBridge	164	63.4%
com.extend.battery	FakeDoc	120	44.1%
com.km.installer	Kmin	65	72.3%
ad.notify1	Opfake	97	96.9%

## Datasets

In this section, we focus on addressing the concern raised in RQ1. We explore three well-known Android malware datasets, Drebin^14^, AMD^18^, and Androzoo^19^ to quantify malware samples sharing the same package names. Table 3 presents the summary of the selected malware datasets. Following is a brief description of the selected datasets:

### Drebin

Drebin dataset was released in 2014 to foster research in the domain of Android malware analysis. Drebin dataset is publicly available and is one of the most cited works in the Android malware domain^20^. Drebin contains 123,453 benign and 5560 malicious apps, including all the apps from Android malware genome project^6^ (one of the pioneer Android malware datasets).

### AMD

Android malware dataset (AMD) was released in 2017 and contains 24,553 Android malware apps belonging to 71 different malware families. AMD consists of malware samples collected from 2010 to 2016 and is one of the largest publicly available Android malware datasets.

### Androzoo

Androzoo is a publicly available, regularly updated and most popular Android apps dataset which is currently being used in recent studies^21,22^. Androzoo was released in 2016 with more than 3 million Android apps and is constantly being updated. By the end of the second quarter of 2021, Androzoo holds more than 15 million Android apps. The Android apps in Androzoo are collected from several platforms like the Google app store, third-party Android app stores and *VirusShare*. The Androzoo dataset’s apps are scanned and labelled for potential malware by using more than 60 antivirus tools. Androzoo provides meta-data for Android apps like size, upload date, signatures and package name in the form of an excel file which is regularly updated. We considered 695,470 malware apps from the Androozoo dataset for analysis of repacked malware based on package names reusing. Our criteria for app selection from Androzoo was that each app must be labelled as malware by at least ten antivirus tools.

**Table 3:** Summary of selected Malware datasets.

Dataset	Families	Samples	Date
Drebin	117	5560	2014
AMD	71	24,553	2017
Androzoo	1969	695,470	2016

**Figure 2:**
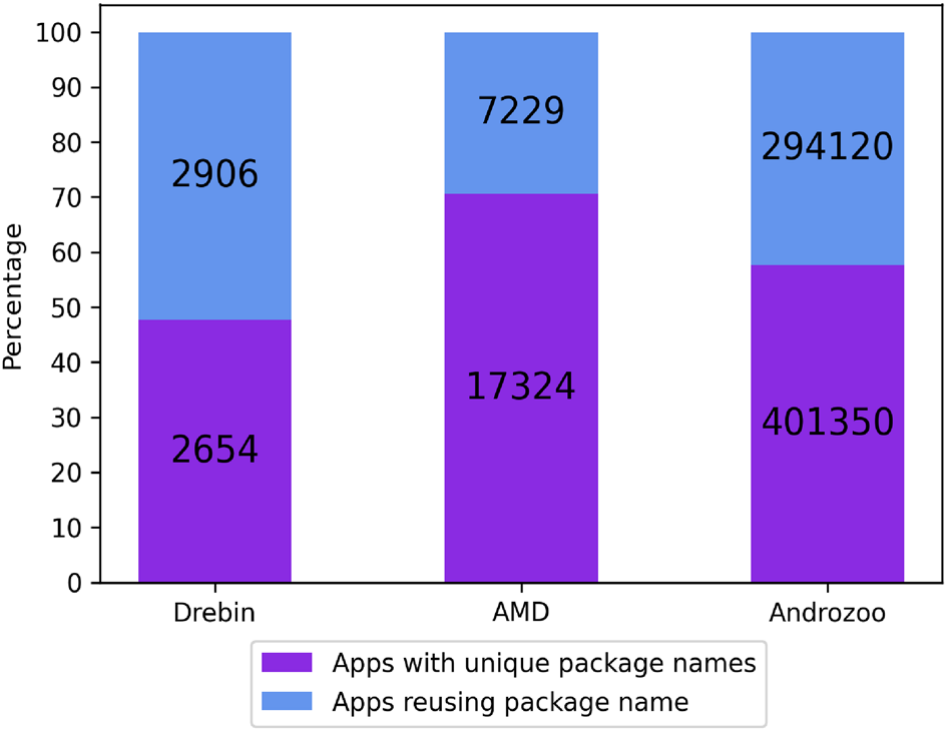
Quantity of Repacked malware in Datasets based on package names reusing.

### Malware clones in datasets

As discussed in Section “Motivation”, our preliminary study on the Drebin dataset revealed the presence of frequently reused package names amongst malware samples. Further investigation on samples sharing the same package names showed that most share almost the same source code. This motivation led us to explore multiple well known Android malware datasets further and quantify the samples sharing the same or similar package names. Although detecting repacked malware based on package names is a lightweight approach and can be easily evaded, our target in this work is to quantify existing clones in the dataset rather than detecting novel clones. The reason is that the selected datasets are very popular amongst the research community, and hence the presence of clones must be considered in future works to avoid biased results. Furthermore, we investigate our claim’s credibility that samples having the same package name are clones of known malware. Based on the results of our initial attempt by using fuzzy hashes it has provided us with the good ground to further investigate by incorporating ML algorithms.

Algorithm 2 presents our methodology to quantify repacked malware in Drebin, AMD and Androzoo datasets based on package names reusing. A dataset is provided as an input to the Algorithm 2, and the number of repacked malware based on reused package names is provided as an output. We take an empty set $P_{names}$ *(Algorithm 2, line 1)* which is populated with the distinct package names in the given dataset. Furthermore, we extract the package names of all the apps in the given dataset using the Androguard tool *(Algorithm 2, line 3)*. AndroGuard^23^ is a python-based tool which can extract multiple features from *AndroidManifest.xml* file of a given APK. The extracted package name is then appended in the Package names list $P_{names}$ if not already present in it *(Algorithm 2, lines 4-6)*. Consequently, the list $P_{names}$ is populated with all the distinct package names within the dataset. The algorithm then returns the number of samples having reused package names (the difference between the number of samples and the number of distinct package names in the dataset).
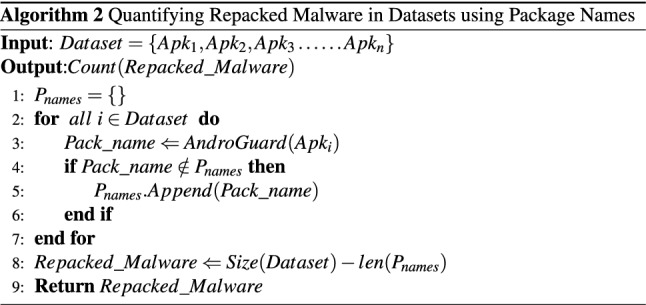


We used the AndroGuard tool to extract package names of samples from Drebin and AMD datasets. In contrast, Androzoo already provides information about the package names. The metadata provided by Androzoo saved a fair amount of time as the Androguard tool performs reverse engineering of an APK to extract features. The time required to reverse engineer an app depends on the size of the app. It took 2.5 seconds on average to reverse engineer apps from Drebin and AMD datasets to extract package names using the Androguard tool (System specification shown in Table 4). Our experiments to find repacked malware samples based on package names reusing in Drebin, AMD and Androzoo datasets are shown in Fig. 2 (addresses RQ1). 52.3% of the samples in Drebin and 29.4% of samples in the AMD dataset contain reused package names. Compared to Drebin and AMD, the Androzoo dataset contains far more samples and interestingly, 42.3% of them contain reused package names. Table 5 outlines the statistics about the top 10 most reused package names in malware samples in each dataset.

**Table 4:** System specifications.

Features	Specifications
Processor	Intel(R) Corei7, 2.60GHz, 6 Cores
GPU	NVIDIA GeForce GTX 1650 Ti 4GB GDDR6
Cache size	12MB
RAM	16 GB DDR4-2933MH
Platform	Windows 10

**Table 5:** Top 10 most reused packages in Datasets.

Drebin		AMD		Androzoo	
Package name	Sample	Package name	Samples	Package name	Samples
com.software.application	234	com.soft.android.appinstaller	548	com.software.application	2114
com.soft.android.appinstaller	193	tk.jianmo.study	384	com.xgbuy.xg	1183
Jk7H.PwcD	117	com.software.application	274	com.soft.android.appinstaller	769
com.extend.battery	110	edu.raj.sphincter	255	ad.notify1	727
ad.notify1	97	jp.bravo.honda	150	com.qihoo.appstore	676
com.convertoman.proin	92	com.android.app	143	ch.nth.android.contentabo_l01_sim_univ	535
vbkoxh.cswnpr	83	org.slempo.service	143	com.nemo.vidmate	475
com.depositmobi	71	fl.affectionate	114	com.qiyi.video	416
com.software.app	54	de.granulocyte	101	nang.dv	408
com.km.launcher	52	org.zxformat	98	tk.jianmo.study	384

## AndroMalPack

**Figure 3:**
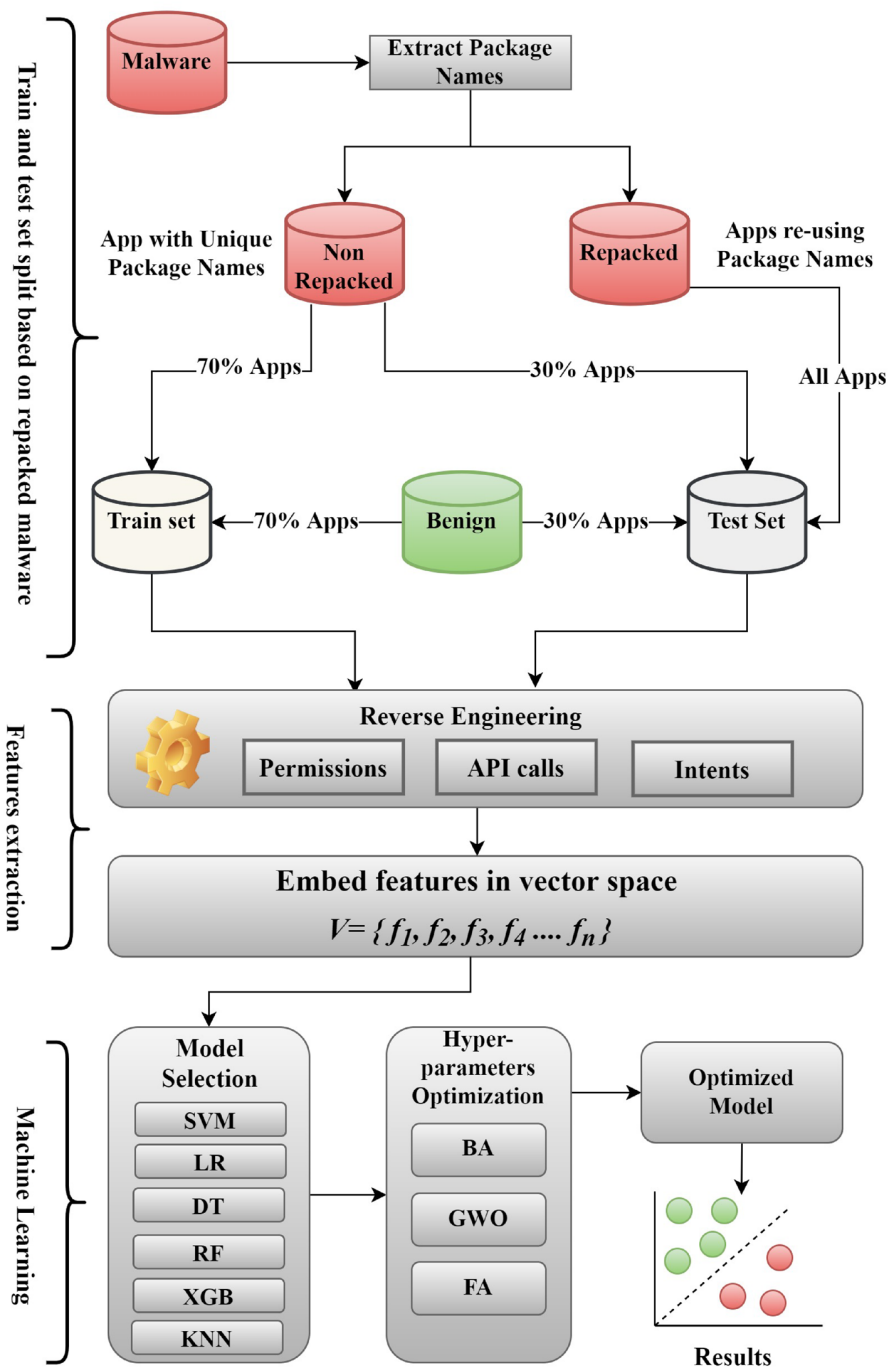
Block Diagram of AndroMalPack.

As discussed in Sect. “Motivation”, signature-based malware detection is very fragile against a simple mutation in original malware. Consequently, malware authors often repack existing malware with minimal modifications to trick antivirus systems relying on signature-based detection. Therefore, we employ ML algorithms to create a more robust solution for repacked malware detection. The motivation to use ML algorithms is to support our claim that malware samples sharing the same package names in popular Android malware datasets are clones of known malware. In this section, we propose AndroMalPack (Fig. 3), an ML-based Android malware classifier trained on clones free train sets and optimized using nature-inspired algorithms (NIAs).

### Data pre-processing

As shown in Fig. 3, AndroMalPack is provided with a malicious Android apps dataset. Instead of splitting the dataset into random train and test sets (the traditional approach), AndroMalPack extracts the apps’ package names to build the train and test sets. All the apps which have reused package names are directly assigned to the test set, whereas 70% of the apps with unique package names are assigned to the train set, and 30% are allocated to the test set. Consequently, train and test set distribution by AndroMalPack confirm the exclusion of malware samples sharing the same package names from the training set and eventually retains diversity and perceptible reduction of training set size. Furthermore, the benign apps dataset apps are randomly distributed 70% in the train set and 30% in the test set.

### Features set modeling

After train and testing set splits, AndroMalPack extracts the features from the Android apps. We use static analysis of apps to extract three different types of features to train ML classifiers. Android permissions and intent filters based features are extracted from *AndroidManifest.xml* file, whereas API calls based features are extracted from the source code of the apps. Following is a brief description of the extracted features:

*Android Permissions* The Android permission model is a framework provided by Android to protect user privacy. It is requisite for an app to acquire permissions from the user before accessing any sensitive features such as sending SMS, using the camera, and accessing contacts and the user’s current location. The pattern of permissions required by an app can be used to train ML algorithms to classify malware and benign apps. Numerous techniques in literature use Android permissions model to detect potential malware in Android apps^2,24,25^.

*Intent filters* define the communication mechanism between different components of an Android app. Intents are simple message objects that transfer the information between different modules such as activities, content providers, services and broadcast receivers of an Android app. The information about intent filters is listed in *AndroidManifest.xml* file and can be used as a feature set to train ML algorithms to classify malware and good-ware apps. Many techniques in literature employ intent filters in addition to other features from *AndroidManifest.xml* file for malware detection^26–28^.

*API calls* Android application programmable interfaces (APIs) are a set of specifications and protocols that are used to build and integrate Android applications. API calls are invoked in apps at run-time to perform different tasks like sending SMS and getting network information. API calls-based features are efficient in malware detection and are used by many existing malware detection techniques^29,30^.
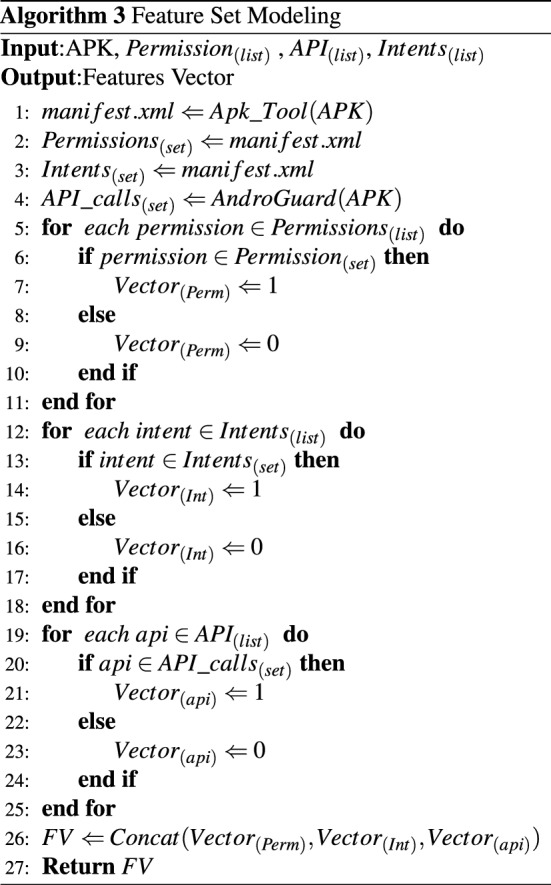


The aforementioned features are employed to construct feature vectors from samples in the datasets. In order to extract the list of distinct permissions, intent filters and API calls, we analysed the *manifest.xml* files and source code of all the Android apps in the three datasets. As a result, we found 623 permissions, 3325 intent filters and 1326 API calls. We further analysed the extracted permissions, intent filters and API calls to build a diverse feature set of discriminating characteristics. We only selected the features with high frequency in malicious and benign apps. Consequently, we build a feature vector containing 308 permissions, 585 intent filters and 226 API calls. We construct a binary encoded feature vector for each APK such that the presence of a particular feature in the APK is marked as 1 in the feature vector whereas absence is marked as 0.

Algorithm 3 explains our methodology for feature set modeling. The Algorithm 3 takes an APK, a list of unique permissions, a list of unique API calls and a list of unique intent filters. APK tool is used to extract *AndroidManifest.xml* file of the given APK *(Algorithm 3, line 1)*. Permissions and intent filters-based features are then extracted from the *AndroidManifest.xml* *(Algorithm 3, line 2-3)*. Furthermore, we use the Androguard tool to extract all the API calls from the given APK *(Algorithm 3, line 4)*. Then we compare each permission in the unique permissions list, and if a particular permission in the unique permissions list is present in the extracted permissions set, the corresponding permissions vector bit is set to 1; otherwise, the bit is assigned 0 value *(Algorithm 3, line 5-11)*. The same process is applied to construct the intents vector *(Algorithm 3, line 12-18)* and the API calls vector *(Algorithm 3, line 19-25)*. Finally, the three vectors (Permissions, intent filters and API calls) are concatenated and returned by the algorithm *(Algorithm 3, line 26-27)*.

### Learning phase

AndroMalPack considers Support vector machines (SVM), Logistic regression (LR), Decision trees (DT), Random Forest (RF), XGBoost (XGB), AdaBoost (AB) and K-nearest neighbours (KNN) to train models. Furthermore, based on the classification results, the best performing model is selected and further tuned using nature inspired algorithms. We consider bat algorithm (BA)^31^, grey wolf optimizer (GWO)^32^ and firefly algorithm (FA)^33^ to optimize the best performing model in motivation to achieve even better classification results. Finally, the results obtained by AndroMalPack are compared with classifiers trained on datasets without considering repacked malware to present the efficacy of AndroMalPack.

## Experimental results

In this section, we report the evaluation results of AndroMalPack. Prompt from the analysis performed in Section “Datasets”, contrary to traditional 80/20 train test splits of datasets, AndroMalPack considers training the classifiers on reduced train sets. The reduced training set of each dataset confirms the exclusion of malware samples sharing the same package names from the training set and eventually retains diversity and perceptible reduction of training set size. Table 6 presents the distribution of samples in train and test sets based on package names from Drebin, AMD and Androzoo datasets. We considered all the samples from Drebin and AMD datasets; however, we contemplated 25116 samples from the Androzoo dataset. As shown in Fig. 2, the Androzoo dataset contains 294,120 potential repacked malware samples, whereas the process of reverse engineering to extract features from all these apps is expensive in terms of time and memory. Therefore, we selected 14,939 samples with unique package names and 10,177 with reused package names from the Androzoo dataset to reduce samples.

**Table 6:** Train and test set splits for classifiers trained on clones free train sets.

Malware dataset	Total malware samples	Malware samples in train set	Benign samples in train set	Malware samples in test set	Benign samples in test set
Drebin	5560	2704	4200	2856	1800
AMD	24553	15157	4200	9396	1800
Androzoo	25116	13039	4200	12077	1800

We evaluate the results based on the outcome of the confusion matrix. Confusion matrix summaries the results of machine learning classifiers based on correct and incorrect predictions by using the following metrics:

- True Positive (TP): signifies the number of malicious apps correctly classified by the ML classifiers.
- False Positive (FP): signifies the number of benign apps classified as malware by the ML classifier.
- True Negative (TN): signifies the number of malicious apps classified as benign by the ML classifier.
- False Negative (FN): signifies the number of benign apps correctly classified by the ML classifier

The performance metrics which we consider are accuracy (Eq. 1), recall (Eq. 2), precision (Eq. 3) and F1-score (Eq. 4) derived from the confusion matrix.

1 $$\begin{aligned} Accuracy = \frac{ TP + TN}{TP + FP + TN + FN} \end{aligned}$$

2 $$\begin{aligned} Recall = \frac{ TP}{TP + FN} \end{aligned}$$

3 $$\begin{aligned} Precision = \frac{ TP}{TP + FP} \end{aligned}$$

4 $$\begin{aligned} F1 = 2 \times \frac{ Precision \times Recall}{Precision + Recall} \end{aligned}$$

**Figure 4:**
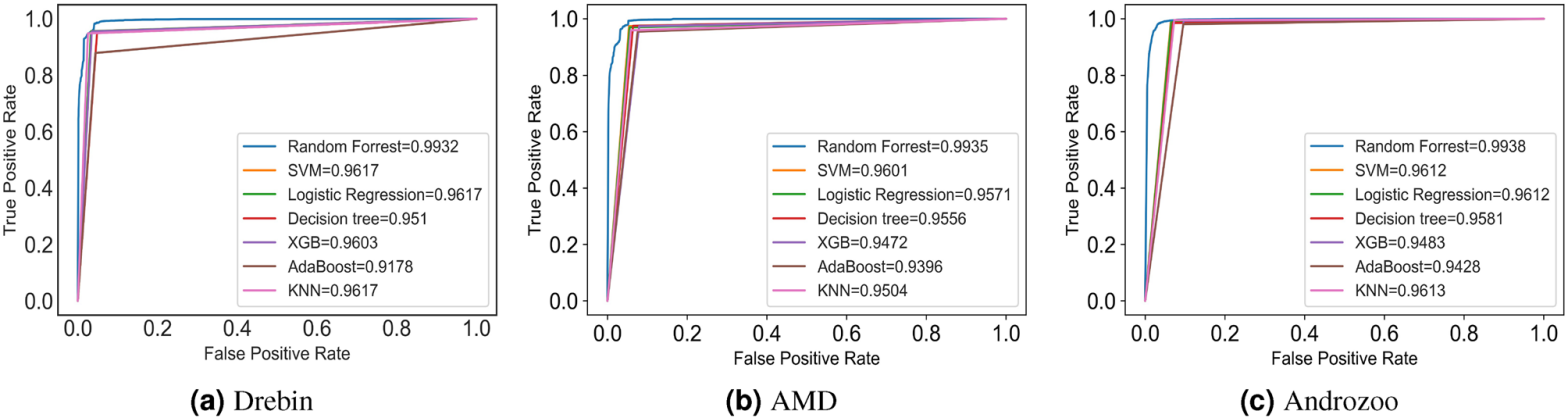
ROC curves of classifiers trained on reduced train sets.

Table 7 presents the results of classifiers trained on reduced train sets with default hyper-parameters settings. Apart from the performance on the Drebin dataset, RF outperforms SVM, LR, DT, AB, XGB and KNN in terms of classification results. Although the classifiers are trained on reduced train sets, whereas test sets contain all the repacked malware samples and non-clone malware and benign apps, RF achieves high precision and recall scores. Similarly, Fig. 4 depicts the receiver operating characteristic (ROC) curves derived from classifiers trained on reduced train sets. The ROC curves plot the false positive rate (FPR) on the x-axis, whereas the true positive rate (Recall) is plotted on the y-axis. The ROC curves show remarkable results where RF yields the best results compared to SVM, LR, DT, AB, XGB and KNN. Subsequently, to further enhance the performance of AndroMalPack, we employ NIAs to determine the optimal hyper-parameters settings of the best performing classifier (RF). We consider Bat algorithm (BA)^31^ (see Appendix A), Firefly algorithm (FA)^33^ (see Appendix B) and Grey wolf optimizer (GWO)^32^ (see Appendix C) for hyper-parameters tuning of RF.

**Table 7:** Results of classifiers trained on reduced train sets.

		SVM	LR	DT	RF	XGB	AB	KNN
Drebin	Accuracy	96.28	96.24	94.88	96.02	96.09	92.27	96.33
	Recall	95.6	95.6	95.4	96.1	95.5	87.9	94.9
	Precision	95.7	95.7	93.1	94.8	95.5	93.9	96.6
	F-measure	95.7	95.7	94.2	95.5	95.5	90.8	95.8
AMD	Accuracy	96.61	96.26	96.43	96.89	95.85	94.61	95.43
	Recall	97.4	97	97.6	97.9	97.4	95.5	96
	Precision	97.7	97.7	97.4	97.8	96.8	96.9	97.6
	F-measure	97.6	97.4	97.5	97.8	97.1	96.2	96.8
Androzoo	Accuracy	97	97.05	96.55	97.53	96.39	95.61	97.31
	Recall	98.7	98.8	98.5	99.5	99.3	98.1	99.5
	Precision	96.9	96.9	96.4	97	97.4	95.5	98
	F-measure	97.8	97.8	97.5	98.2	97.4	96.8	98

**Figure 5:**
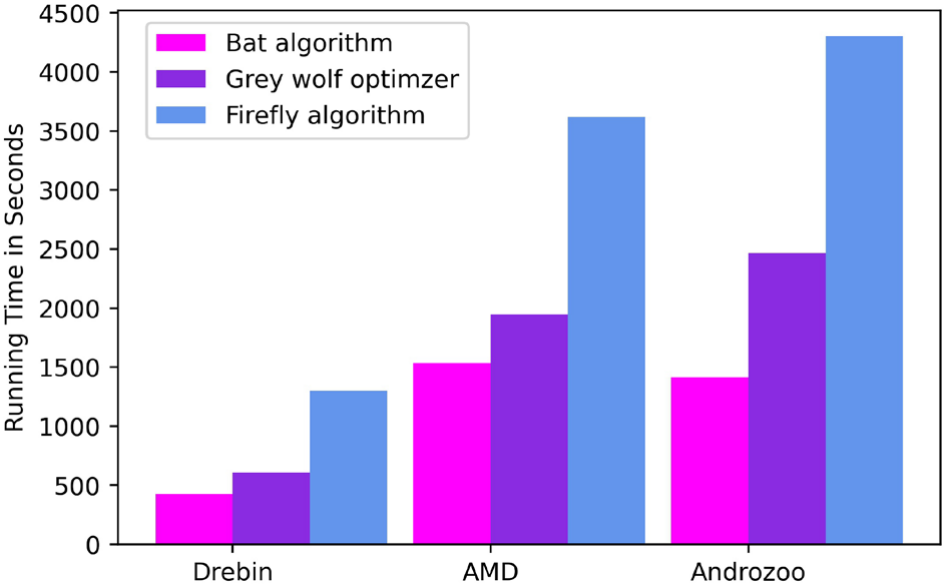
Running time comparison of NIAs.

Table 8 present the optimal hyper-parameters setting for RF classifiers determined by NIAs (BA, FA and GWO) based on Drebin, AMD and Androzoo datasets. Furthermore, Table 9 presents the classification results achieved by AndroMalPack, an Android malware classifier based on RF and optimized using NIAs. As compared to the results of the RF classifier in Table 7, AndroMalPack remarkably strengthens the performance by employing NIAs to determine the optimal setting of hyper-parameters. Furthermore, as shown in Table 9, in the case of each dataset, RF optimized using BA performs slightly better than FA and GWO, whereas the result obtained from FA and GWO are almost similar with a marginal difference. However, in addition to classification results, we also consider the time complexity of NIAs as a performance metric for AndroMalPack. Figure 5 depicts the time taken by each NIA (BA, FA and GWO) for optimizing the hyper-parameters of RF-based on Drebin, AMD and Androozoo datasets. The population size for each NIA was initialized with 50, and max iterations were set to 100. Subsequently, BA outperforms FA and GWO in terms of time complexity and classification results. Nevertheless, the performance of FA and GWO is also convincing in terms of classification results; however, as compared to BA, FA and GWO take a significant amount of time to find the optimal hyper-parameters in the case of each dataset (Fig. 5). Therefore, AndroMalPack prefers BA compared to FA and GWO for hyper-parameters optimization to enhance the performance of RF for Android malware classification.

**Table 8:** Hyper-parameters for RF proposed by NIAs.

	*n_estimators*			*max_depth*			*min_sample_split*			***max_features***		
	BA	FA	GWO	BA	FA	GWO	BA	FA	GWO	BA	FA	GWO
Drebin	60	80	80	34	28	28	2	2	2	*auto*	*sqrt*	*auto*
AMD	60	80	80	36	38	38	2	2	2	*sqrt*	*sqrt*	*sqrt*
Androzoo	40	80	80	32	32	32	2	2	2	*sqrt*	*sqrt*	*auto*

**Table 9:** Results of AndroMalPack.

	Bat Algorithm				Firefly Algorithm				Grey Wolf Optimzer			
	Acc	Recall	Pre	F1	Acc	Recall	Pre	F1	Acc	Recall	Pre	F1
Drebin	98.29	98.7	97.7	98.1	98.22	98.5	97.4	98	98.22	98.5	97.4	98
AMD	98.21	99.4	98.1	98.7	98.17	99.4	98.1	98.7	98.17	99.4	98.1	98.7
Androzoo	97.94	99.8	97.2	98.5	97.9	99.8	97.2	98.5	97.94	99.8	97.2	98.5

**Table 10:** Results of classifiers on Datasets using Random 80/20 Train and Test Splits.

		SVM	LR	DT	RF	XGB	AB	KNN
Drebin	Accuracy	96.03	96.25	95.74	97.09	95.78	94.98	97.6
	Recall	94.5	94.7	95.9	96.8	94.2	92.2	96.8
	Precision	95.5	95.8	93.6	95.9	95.1	95.1	97.1
	F-measure	95	95.3	94.7	96.4	94.7	93.6	97
AMD	Accuracy	97.71	97.81	97.59	98.27	97.56	95.86	98.04
	Recall	98.6	98.7	98.5	99.5	99.1	97.3	99.3
	Precision	98.4	98.3	98.3	98.2	97.7	97.2	98.1
	F-measure	98.5	98.5	98.4	98.8	98.4	97.2	98.7
Androzoo	Accuracy	97.38	97.38	97.29	98.26	96.75	95.83	97.8
	Recall	98.8	98.7	98.6	99.6	98.9	97.8	99.2
	Precision	97.7	97.7	97.7	98.1	96.7	96.6	97.8
	F-measure	98.2	98.2	98.2	98.8	97.8	97.2	98.5

The experimental results show that although AndroMalPack excludes all the repacked malware (based on package name reusing) from training sets, whereas test sets contain all repacked malware in addition to non-repacked and benign samples, it achieves a remarkable detection accuracy (up to 98.2%). Therefore our experiments address the **RQ2** by proving that the malware samples sharing the same package names can be considered repacked malware. Furthermore, we consider the traditional 80/20 random train test split regardless of repacked malware in the datasets to compare the results with AndroMalPack. Table 10 shows the classification results obtained from classifiers based 80/20 train and test sets split with 10-fold cross-validation. Apart from the classification results from the Drebin dataset, the RF classifier outperforms all the other classifiers in terms of accuracy, recall, precision and F1 score. Compared to the classification results of AndroMalPack, the results obtained by classifiers based on 80/20 random train test splits are subtle with a marginal difference. Consequently, these experiments address the **RQ3** as we can conclude that removing repacked malware based on the same package names from training sets does not significantly affect the classification results of ML-based algorithms.

## Discussion

To prove that malware samples sharing the same package names are repacked versions of known malware, AndroMalPack assigns all samples with reused package names to the test set in addition to benign apps and non-repacked malware. Interestingly, AndroMalPack achieves up to 98% accuracy with the train and test set distribution. The results reflect our claim that malware samples sharing the same package names are clones of existing malware. The analysis of the datasets (Drebin, AMD and Androzoo) reveals that numerous malware samples in these repositories are repacked (based on package name reusing). We emphasize that repacked malware should be of concern while performing Android malware analysis. Repacked malware creates an overhead in terms of time and computational expenses. Hence, removing the repacked malware can save a fair amount of time in the reverse engineering process to extract features from Android apps.

**Figure 6:**
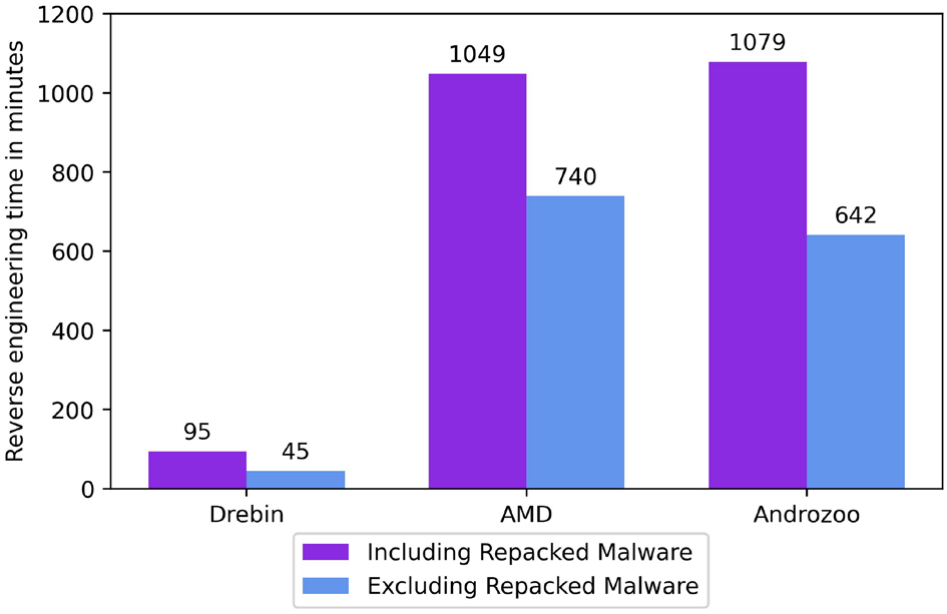
Features extraction time comparison.

In order to present the effectiveness of removing repacked malware from the datasets, we profile the reverse engineering time to extract features based on two scenarios. In scenario 1, we consider reverse-engineering the full dataset regardless of repacked malware, whereas, in scenario 2, we remove the repacked malware and profile the reverse engineering time. As shown in Fig. 6, removing repacked apps in Drebin, AMD and Androzoo datasets significantly reduced the processing time. It took, on average, 2.5 s to extract APIs, intents, and permissions-based features from an APK by employing the Androguard tool’s static analysis. On the other hand, dynamic analysis can take anywhere between 60 s and 10 min per APK to extract features^34–37^.

Nevertheless, the evaluation results of AndroMalPack prove that removing the repacked malware from training sets does not significantly impact classification results. Furthermore, as discussed in^38^ and^39^, the duplicates in datasets can cause adverse effects on ML models by producing biased results. Consequently, we encourage fellow researchers to consider repacked malware in Android malware datasets while performing ML-based malware detection to train classifiers on reduced yet diverse data. Moreover, in addition to automated analysis, malware analysts generally perform manual dissection of malicious apps to study the insights of malware. Since our experiments show that the malware samples sharing the same package names are repacked versions of known malware, industrial specialists can employ our technique as a first-order pruning mechanism for malware analysis to save time and expense. Moreover, in this work, we have shown that signature-based techniques are vulnerable to detecting repacked malware. Since we published a dataset comprising 389,995 repacked Android apps which reuse existing package names, the industry can leverage it to develop novel and more robust signature generation techniques with the ability to detect repacked malware.

## Related work

### Android malware detection

Malware has seen significant development in recent years, making it more complex than ever. Malware has impacted domains as diverse as cloud services, smart grids, financial institutions, and cryptocurrency mining^40–42^. Due to the widespread usage of Android OS-based smart devices (70% market share in the mobile OS industry), they have become a prime target for malware developers. As a result, the research community has expressed a significant interest in securing Android devices against malicious attacks^43,44^. Many researchers have demonstrated machine learning as the core element of Android malware detection. Drebin^14^, one of the most cited works in the Android malware detection domain, employed a characteristics-based method for Android malware detection. Drebin performed static analysis to extract multiple features from Android apps such as APIs, permissions, intents and hardware components to train a linear SVM model to classify malicious and benign apps. The evaluation results of Drebin report 94% malware detection accuracy with a meagre false positive rate.

Ali Feizollah et al.^45^ proposed Androdialysis, a technique to detect Android malware using intents-based features. The technique suggests that intents are semantically rich features to detect malware with more detection accuracy than permissions-based features. They evaluated the Androdialysis using the Drebin dataset (5560 malicious 1846 benign apps) and achieved up to 91% malware detection accuracy. Garcia et al. proposed RevealDroid^46^, an Android malware detector based on a large spectrum of static feature space. RevealDroid claims to achieve detection accuracy up to 98% on a dataset comprising 54000 malicious and benign Android apps. Surendran et al.^47^ proposed GsDroid, a technique to represent Android apps as a directed graph of sequenced system calls and combined ML-based algorithms to learn from malicious patterns. GsDroid obtained up to 99% malware detection accuracy on various Android malware datasets. Maryam et al. proposed cHybriDroid^48^, an Android malware classifier based on the conjunction of static and dynamic features of Android apps. They employed tree-based pipeline optimization technique (TPOT)^49^ to formulate a malware detection model and achieved up to 96% malware detection accuracy on the Drebin dataset. Pye et al.^50^ proposed a framework to detect Android malware using ML-based techniques. They optimized various ML algorithms using nature-inspired algorithms and achieved up to 99.6% malware detection accuracy.

Bai et al. proposed a siamese network-based learning technique to classify Android malware families^51^. Apart from large malware families, Bai et al. significantly improved the detection accuracy of few shot malware families. Fan et al.^52^ employed a graph-based method to construct frequent sub-graphs based on API calls to identify common behaviour between the same Android malware families. Similarly, Frenklach et al.^53^ extracted application similarity graphs based on function calls and combined ML algorithms to detect Android malware. Consequently, they achieved up to 95.5% accuracy on various Android malware datasets. Hongyu and Tang^54^ considered the power consumption of Android applications as a feature to detect malware. They profiled the power consumption of different categories of apps, where each app was monitored for 5 minutes. Based on the profiled data, they were able to detect 79 out of 100 malicious Android apps in the test set. Although ML-based malware detection techniques have demonstrated high classification accuracy, however, these techniques are vulnerable to evasive malware. Malware is swiftly evolving to evade the current countermeasures which are proposed in literature and used in commercial antivirus tools^55^. Therefore many recent Android malware techniques focus not just to accurately classify Android malware but also to counter evasion attacks. Rafiq et al.^56^ presented the fragility of Android malware classifiers in adversarial settings. They proposed a cumulative adversarial training scheme to counter the evasion attacks on ML-based Android malware classifiers and demonstrated a 99.46% detection of evasive Android malware. Salman et al.^57^ used GANs to harden the security of Android malware detectors against evasion attacks through intents based features. Similarly, the authors in^58^ claim that GAN based methods improve the evasion detection of Android malware up to 50%.

### Android malware repackaging

Android malware repackaging has become a significant concern for security analysts over the past few years. Currently, most antivirus systems rely on the signature-based detection^5,59,60^. In contrast, application repackaging or creating clones of Android malware have become a common practice by attackers to evade such techniques. During the past few years, the research community have shown prevalent interest in the detection of repacked and cloned malware by employing alternative techniques^8^. Zhou et al. presented one of the preliminary studies on repacked malware in the Android malware domain and claimed that more than 80% of the existing Android malware is repacked^6^. Likewise, DNADroid^61^ was proposed to detect potential clones of Android apps by using dependency graphs based on methods in the Android app. Zheng et al. proposed DroidAnalytics^62^, an Android malware detector based on a multi-level signature generation technique with the ability to determine malware clones. ImageStruct^63^ and a similar work DroidEagle^64^ leverage the similarity of images and UI layout to detect potential clones and repacked malware in Android apps. DroidClone^65^ rely on the structure and reusing of code segments to detect repackaged apps and clones of Android malware. Singh et al. employ a multi-view machine learning-based technique to detect repacked Android malware^66^ and report up to 97.46% accuracy using 15,297 malware samples.

Glanz et al. proposed CodeMatch^67^, a technique based on advanced library detection and fuzzy hashing to detect repacked Android apps. They applied the CodeMatch tool on various Android app stores and revealed that 15% of the apps in the commercial app stores are repacked versions of known apps. Ishii et al.^68^ proposed Appraiser to perform a large-scale analysis of cloned apps in Android app repositories. They evaluated 1.3 million apps from various Android app stores and found that around 13% of the apps in third-party app stores are clones of existing apps. Furthermore, they revealed that up to 70% of the cloned apps in third-party app stores are repacked versions of known malware. Gaofeng et al.^69^ proposed a technique to detect repacked Android malware based on mobile edge computing. They employed the Density Peak Cluster method on network traffic data to find the similarities between Android apps. As a result, they detected up to 92% of the repacked apps in the dataset. Alam et al.^70^ proposed DroidClone to address the problem of clones in Android malware. DroidClone employs *MAIL*, a novel language to identify control flow patterns in the program. When evaluated on a dataset of 2050 malware and 2130 benign Android apps, DroidClone achieved a detection rate of up to 94.2%. A recent study by Roopak Surendran^71^ investigated the impact of semantically similar Android malware apps on various ML models. Surendran employed an opcode subsequence-based clustering technique to identify malware clones in the Drebin dataset. The results show that the malware detection rate drops from 95% to 91% when malware clones are removed from the dataset.

This work focuses on a simple yet powerful strategy for repacked malware detection by using package name-based similarity. We demonstrated that many apps in popular Android malware repositories share common package names. Our further analysis revealed that apps sharing the same package names are repackaged versions of existing malware. Similarly, most of the existing techniques focus on the detection of repacked and cloned malware using various techniques and report that plethora of malware is repacked instead of being novel^8^. However, in the Android malware domain, apart from the study proposed by Zhao et al.^39^, no extensive study has been conducted on the impact of duplicates on ML classifiers. Zhao et al. considered duplicates based on three distinct features (*.dex* code similarity, op-code sequence and API calls). They evaluated them using four different datasets (Genome, Drebin, AMD and RmvDroid^72^). Compared to Zhao et al., we considered a novel and more lightweight strategy (package names based on similarity). Interestingly, in the case of the Drebin dataset, package names-based similarity (52.3%) outperforms, *.dex* code similarity (35.9%) and op-code sequence (48.6%) in^39^ to detect malware clones, whereas API based similarity is almost similar to our approach (52.4%). However, in the case of the AMD dataset, apart from *.dex* code similarity (21.8%), Op-code (47.6%) and API calls based similarity (52.2%) outperforms package based similarity (29.4%). Likewise, Irolla et al. use op-code similarity to quantify duplicates in Drebin dataset^73^. Irolla et al. claim that 49.35% samples in the Drebin dataset are repackaged and question the biased results of existing ML classifiers trained on the Drebin dataset. As compared to^73^, package name based repackaged malware detection is more lightweight and outperforms Irolla et al. technique by finding 52.3% repacked malware in the Drebin dataset.

Furthermore, we propose AndroMalPack, an Android malware classifier trained on clones free training sets and optimized using NIAs. The training sets of AndroMalPack exclude all the apps which share common package names and consequently reduce the size of training data yet preserve high classification results. Table 11 presents the detailed comparison of AndroMalPack with recent Android malware detection techniques in literature.

**Table 11:** Comparison of AndroMalPack with related work.

Technique	ML-Model	Features	Dataset	Accuracy	Recall	Precision
^74^	Ensemble	Permissions and source code	M0Droid	95.6	95.7	95.8
^75^	Random Forrest	Permissions, APIs and system calls	Custom	88.26	88.40	88.16
^76^	Random Forrest	Permissions, APIs,intents and hardware	Drebin	97.24	96.88	97.58
^77^	Random Forrest	API calls	Drebin	96	95	97
			AMD	98	98	99
^78^	CatBoost	Permissions and op-code sequence	Drebin and Custom	97.40	96.77	98.0
^79^	Random Forrest	Permissions based features	Androzoo	81.53	81.53	82.59
^80^	Profile hidden Markov model	API calls	Drebin	94.5	N/A	93.0
^81^	SVM	Permission, intents and byte-code	AMD	95.09	95.09	95.11
**AndroMalPack**	Random Forrest	Permission, API calls and Intents	Drebin	98.29	98.70	97.7
			AMD	98.21	99.40	98.1
			Androzoo	97.14	99.8	97.2

## AndroMalPack dataset

In order to foster the research in the domain of repackaged Android malware analysis, we publish a cryptographic hash-based dataset of repacked Android apps having the same package names (AndroMalPack dataset). AndroMalPack dataset is distributed into three comma-separated (*.csv*) files where each file contains cryptographic hashes of repacked apps from Drebin, AMD and Androzoo datasets, respectively. Each file in the AndroMalPack dataset contains two columns where the first column contains the hash of the app and the second column contains the corresponding package name. The files are sorted in descending order based on the number of frequently reused package names in each dataset. Since the access to Drebin, AMD and Androzoo are protected by the owners, we do not provide the APK files. Access to the datasets (Drebin, AMD and Androzoo) can be requested through an authorized source, and our dataset of hash values can be employed to churn out repackaged apps based on package name reusing. Drebin and Androzoo datasets label each app with a SHA256 hash, whereas AMD datasets label apps using MD5 hashes. Likewise, the AndroMalPack dataset uses SHA256 hashes for Drebin and Androzoo, whereas MD5 hashes for the AMD dataset to represent repackaged apps based on package name reusing.

## Conclusion

Malware authors often repack existing malware to deceive antivirus systems, due to which numerous apps in popular Android malware datasets are clones of existing malware. This paper emphasizes the problem of repacked Android malware in benchmark Android malware repositories. To identify repacked malware, we employed a novel and lightweight strategy of matching the package names of malware samples with known malicious package names. As a result, we found that 52.3% malware samples in Drebin, 29.8% of malware samples in AMD and 42.3% malware samples in the Androzoo dataset reuse existing package names. Furthermore, we proposed AndroMalPack to support our claim that the apps sharing the same package names are clones of known malware. Contrary to the traditional 70/30 train and test set split, AndroMalPack assigns all samples with reused package names to the test set in addition to benign apps and non-repacked malware. Our experiment results present that although AndroMalPack is trained on reduced train sets, it preserves a remarkable malware detection accuracy of up to 98%. Furthermore, we demonstrated that the presence of malware clones in the datasets causes overhead in terms of time and resource expenses and does not significantly impact the results of ML-based malware classifiers. Finally, we publish an AndroMalPack dataset to foster the research on repackaged Android malware based on package names reusing. AndroMalPack dataset contains 389,995 cryptographic hashes of samples sharing the same package names in the Drebin, AMD and Androzoo datasets.

## Supplementary Information

Supplementary Information.

## Data Availability

This study investigates Drebin, AMD and Androzoo datasets to quantify repacked Android malware. Since the access to these datasets is protected by the owners, we do not provide the APK files. Access to the datasets (Drebin, AMD and Androzoo) can be requested through an authorized source, and AndroMalPack dataset of hash values can be employed to churn out repackaged apps based on package names reusing.
