# Supplementary material for: AndroMalPack: enhancing the ML-based malware classification by detection and removal of repacked apps for Android systems
Source: Sci Rep. 2022 Nov 14;12:19534. doi: 10.1038/s41598-022-23766-w (PMC9663591; doi:10.1038/s41598-022-23766-w)
Supplement: Supplementary file 1 — Supplementary Information. [file 41598_2022_23766_MOESM1_ESM.pdf]

Appendix A Bat Algorithm

The bat algorithm is inspired by the echolocation behaviour of micro bats. Bats have extraordinary capability to find prey, avoid obstacles and differentiate between insects in the dark by using sonar. BA is formulated by mimicking the characteristics of bats based on the following rules:

- Bats use echolocation to estimate distance, find prey, avoid obstacles and distinguish between target by nature.
- Bats fly at a random velocity v_i at position x_i with fixed frequency f_{min}, varying wave lenghts λ and loundness A_o in order to search for potential prey. Bats have the capability to automatically adjust the wavelength (or frequency) of their emitted pulse and can adjust the pulse emission rate $r \in [0,1]$ depending on the position of target.
- The loudness of Bats can vary from a large value A_o to a constant minimum value A_{min}.

Based on the aforementioned rules, BA algorithm is presented in Algorithm 1.

Algorithm 1 Bat Algorithm

Input: The objective function $f(x)$, $x = \{x_1, x_2, x_3 \dots x_d\}$
Output: Optimal position of bat x_*

Initialize the bat population at position x_i
$i = \{1, 2, 3 \dots n\}$
Define pulse frequency f_i at s_i
initialize pulse rate r_i and loudness A_i
while $t < max\_iterations$ **do**
    generate new solutions by adjusting frequency f_i, updating velocity v_i and location x_i
    if $rand > r_i$ **then**
        select a solution among the best solutions
        generate a local solution based on the selected solution
    end if
    generate a new solution by flying randomly
    if $rand < A_i$ and $f(x_i) < f(x_*)$ **then**
        Accept the new solutions
        Increase r_i and A_i
    end if
    Rank the bats and find the current best x_*
end while
Return x_*

Appendix B Firefly Algorithm

FA is a meta-heuristic algorithm for optimization problems inspired by the flashing patterns and behavior of fireflies. Firefly algorithm is formulated by using following three rules:

- Fireflies are attracted to other fireflies based on the intensity of their brightness.
- The attractiveness and brightness of firefly decreases as it moves away from other fireflies. Fireflies start to move randomly if they are unable to find a brighter firefly.
- An objective function is used to determine the brightness of a particular firefly.

Based on the aforementioned rules, the pseudo code of FA is presented in algorithm 2.

Appendix C Grey wolf optimizer

GWO is a meta-heuristic algorithm inspired by the social hierarchy and hunting strategy of grey wolves. Grey wolves live in a pack of 5 to 12 and are divided in to 4 different classes (alpha, beta, delta and omega) based on individual responsibilities. Alpha wolf is the head of the pack (regardless of gender) and is responsible to organize, make decisions and lead the pack. Beta wolf is second to the superior in the pack. It assists alpha wolf in decision making and has the authority to take over the command in case of injury or senility of alpha wolf. Delta wolves are the scouts and have the responsibility for security and

Algorithm 2 Firefly Algorithm

Input: The objective function $f(x)$, $x = \{x_1, x_2, x_3 \dots x_d\}$
Output: brightest fireflies

```
Initialize the fireflies population x_i, i = {1,2,3...n}
Define light the Intensity I based on f(x)
Define the absorption coefficient γ
while t < max_Generation do
    for i = 1 : n (all n fireflies) do
        for j = 1 : i (n fireflies) do
            if I_j > I_i then
                move firefly i toward firefly j
            end if
            Vary attractiveness with distance r via exp(−γr)
            Evaluate new solutions and update light intensity
        end for
    end for
    Rank fireflies and find the current best
end while
Return brightest fireflies
```

hunting activities for the pack. Finally, Omega wolves are the elders or the frail wolves. Mostly, they have the responsibility to take care of the off springs. Grey wolves are known for their extraordinary technique for hunting by employing following 3 steps:

- Track, tail and approach towards the prey.
- Encircle, harass and move towards the prey until it becomes to a stationary state.
- Simultaneously attack the prey .

Equation 1 shows the mathematical representation of encircling the prey characteristics of grey wolves.

$$X(t+1) = X_p(t) - A.\|C.X_p(t) - X(t)\| \tag{1}$$

where X represents the position of the wolf, the current iteration is presented by t and X_p is the current location of the prey. The controlled coefficients A and C are calculated with the help of equation 2 and equation 3

$$A = 2a.r_1 - a \tag{2}$$

$$C = 2a.r_2 \tag{3}$$

where r_1 and r_2 are randomly generated during iterations from a range of $[0,1]$ respectively. The controlled vector a linearly decreases from 2 to zero during the iterations as shown in equation 4.

$$a(t) = 2 - 2.(t/T) \tag{4}$$

where T represents the maximum number of iterations. The other wolves in the pack update their position based on the position of alpha (α), beta (β) and delta (δ) wolves as shown below:

$$D_\alpha = \|C_1 \times X_\alpha - X\| \tag{5}$$

$$D_\beta = \|C_2 \times X_\beta - X\| \tag{6}$$

$$D_\delta = \|C_3 \times X_\delta - X\| \tag{7}$$

$$X_1 = X_\alpha - A_1.D_\alpha \tag{8}$$

$$X_2 = X_\beta - A_2.D_\beta \tag{9}$$

$$X_3 = X_\delta - A_3.D_\delta \tag{10}$$

$$X(t+1) = \frac{X_1 + X_2 + X_3}{3} \tag{11}$$

where the distance of current wolf from α, β and δ is represented by Equation 5, Equation 6 and Equation 7 respectively. Consequently, Equation 8, Equation 9 and Equation 10 present the position of remaining grey wolves based on the current location of α, β and δ. Finally, Equation 11 is used to calculate the updated the position of the wolf. Based on the aforementioned explanation, the pseudo-code of GWO is presented in Algorithm 3.

Algorithm 3 Grey wolf optimizer Algorithm

Input: The objective function $f(x)$, $x = \{x_1, x_2, x_3 \ldots x_d\}$
control coefficient a
number of iterations
Output: Optimal position of X_α

```
Initialize the grey wolves population x_i, i = {1,2,3...n}
Identify X_α, X_β and X_δ based on objective function
while t < max_Iterations do
    for each grey wolf in pack do
        compute A and Cby Eq. 2 and 3
        Update the position of current wolf using X_α, X_β and X_δ by Eq.11
    end for
    Update a, A and C
    Calculate the fitness of each wolf
    Update X_α, X_β and X_δ
end while
Return Best solution X_α
```
